# Comparative Effects of Human Neural Stem Cells and Oligodendrocyte Progenitor Cells on the Neurobehavioral Disorders of Experimental Autoimmune Encephalomyelitis Mice

**DOI:** 10.1155/2016/4079863

**Published:** 2016-06-26

**Authors:** Dae-Kwon Bae, Dongsun Park, Sun Hee Lee, Goeun Yang, Jangbeen Kyung, Dajeong Kim, Kyungha Shin, Ehn-Kyoung Choi, Gonhyung Kim, Jin Tae Hong, Seung U. Kim, Yun-Bae Kim

**Affiliations:** ^1^College of Veterinary Medicine, Chungbuk National University, Cheongju 28644, Republic of Korea; ^2^College of Pharmacy and Medical Research Center, Chungbuk National University, Cheongju 28644, Republic of Korea; ^3^Department of Medicine, University of British Columbia, Vancouver, BC, Canada V6T 2B5

## Abstract

Since multiple sclerosis (MS) is featured with widespread demyelination caused by autoimmune response, we investigated the recovery effects of F3.olig2 progenitors, established by transducing human neural stem cells (F3 NSCs) with Olig2 transcription factor, in myelin oligodendrocyte glycoprotein- (MOG-) induced experimental autoimmune encephalomyelitis (EAE) model mice. Six days after EAE induction, F3 or F3.olig2 cells (1 × 10^6^/mouse) were intravenously transplanted. MOG-injected mice displayed severe neurobehavioral deficits which were remarkably attenuated and restored by cell transplantation, in which F3.olig2 cells were superior to its parental F3 cells. Transplanted cells migrated to the injured spinal cord, matured to oligodendrocytes, and produced myelin basic proteins (MBP). The F3.olig2 cells expressed growth and neurotrophic factors including brain-derived neurotrophic factor (BDNF), nerve growth factor (NGF), ciliary neurotrophic factor (CNTF), and leukemia inhibitory factor (LIF). In addition, the transplanted cells markedly attenuated inflammatory cell infiltration, reduced cytokine levels in the spinal cord and lymph nodes, and protected host myelins. The results indicate that F3.olig2 cells restore neurobehavioral symptoms of EAE mice by regulating autoimmune inflammatory responses as well as by stimulating remyelination and that F3.olig2 progenitors could be a candidate for the cell therapy of demyelinating diseases including MS.

## 1. Introduction

Multiple sclerosis (MS) as an autoimmune-mediated neurological disease frequently occurs in young adults in the Western countries. The MS patients suffer from neurobehavioral disorders caused by demyelination and neuronal damage [1]. Thus, it is believed that early pathological features of MS are characterized as widespread axonal demyelination following the injury of myelin-producing oligodendrocytes. In experimental autoimmune encephalomyelitis (EAE), an animal model of MS, autoimmune Th1 cells activated by injected myelin antigens release cytokines including interferon-*γ* (IFN-*γ*) and interleukin-2 (IL-2) which attract the macrophages into the central nervous system (CNS) and induce the inflammation [2–4]. Although remyelination after demyelination is a common spontaneous repair process, most of patients with consistent demyelination and axonal loss after failure of the recovery process progress to irreversible functional deficits in CNS [5].

Immunomodulatory and neurotrophic factors which have been currently used for treatment of MS are ineffective under irreversible conditions because those are impossible to regenerate the severely damaged myelins [6]. In contrast, recent studies revealed that stem cells improved even the irreversible CNS defects via cell replacement, neuroprotection, and immunoregulation mechanisms [6, 7]. For example, intravenous transplantation of neural stem cells (NSCs) to EAE animals alleviated the clinical symptoms by modulating the peripheral immunity [3] and intracerebroventricularly transplanted NSCs improved the CNS disorders through replacement of the damaged cells and protection of the host cells [8].

Also, immortalized F3 human NSCs revealed multipotent capacity to differentiate into neurons and glial cells [9–11] and replaced defected areas in animal models of stroke [12, 13]. However, differentiation of F3 NSCs into oligodendrocytes was very low both* in vitro* and* in vivo* [14]. In addition, it was demonstrated that other human stem cells also have a little ability of differentiation into the oligodendrocytes [15–17]. Although the mechanism is not well understood, it is obvious that oligodendrocytes are the main autoimmune target cells in MS; that is, excessive degeneration of oligodendrocytes leads to progressive demyelination, inducing delayed neuronal conduction [18], while glial cell transplantation and induced differentiation of oligodendrocyte precursor cells promoted remyelination of EAE animals [8, 16]. Therefore, the protection and regeneration of oligodendrocytes might be an important therapeutic strategy for MS.

Notably, it was reported that transduction of F3 human NSCs with Olig2 transcription factor led to exclusive differentiation into O4- and CNPase-positive oligodendrocyte progenitors (F3.olig2), activation of Nkx2.2, and proliferation with overexpressed Olig2* in vitro* [14, 19, 20]. Moreover, transplantation of the human F3.olig2 progenitor cells into spinal cord injury (SCI) rats facilitated regeneration of myelins in the white matter of spinal cords and recovery of locomotor activity, which was superior to F3 NSCs [14]. In addition, it was confirmed that the F3.olig2 cells expressed and released growth and neurotrophic factors responsible for neuroprotective and regenerative activities as in F3 cells. In the present study, recovery effects of F3.olig2 human oligodendrocyte progenitor cells on the neurobehavioral disorders of EAE model animals were investigated based on the action mechanisms, in comparison with their parental F3 neural stem cells.

## 2. Materials and Methods

### 2.1. Establishment of F3.olig2 Human Oligodendrocyte Progenitors

An immortalized human NSC line, HB1.F3 (F3), was established from primary cultures of a 15-week gestational human fetal brain by infecting with a retroviral vector encoding v-*myc *oncogene [9, 21]. To generate Olig2 overexpressing oligodendrocyte progenitor cells, Olig2 cDNA (a generous gift from Dr. Takebayashi, Okazaki, Japan) was ligated into multiple cloning sites of the retroviral vector pLPCX. PA317 amphotropic packaging cell line was infected with the recombinant retroviral vector, and the supernatants from the packaging cells were added to the F3 cells. Stably transfected colonies were selected by puromycin resistance, and the immortalized F3.olig2 oligodendrocyte progenitor cell line exclusively differentiated into O4- and CNPase-positive oligodendrocytes, overexpressing Olig2* in vitro* [14, 19].

### 2.2. EAE Induction and Cell Transplantation

Six-week-old male C57B/6 mice (*n* = 10/group) were purchased from a commercial breeder (Daehan Biolink, Eumseong, Korea). They were housed in a room with constant temperature (23 ± 3°C), relative humidity (50 ± 10%), and 12-hour light cycle. Animals were fed a standard commercial rodent chow (Daehan Biolink).

EAE was induced in 7-week-old mice by subcutaneous immunization with an emulsion containing 300 *μ*g purified MOG peptide (Ana Spec Inc., Fremont, CA, USA) in 300 *μ*L PBS and 1.2 mg* Mycobacterium tuberculosis* (stain H37RA; Difco, Detroit, MI, USA) in the same volume of complete Freund's adjuvant. In addition, 300 ng* Bordetella pertussis* toxin (Sigma-Genosys, Cambridge, UK) in 0.1 mL PBS was intravenously injected on days 0 and 2. The clinical scores of EAE were recorded daily for up to 50 days as follows: grade 0 = asymptomatic; grade 1 = partial loss of tail tonicity; grade 2 = atonic tail; grade 3 = hind leg weakness and/or difficulty to roll over; grade 4 = hind leg paralysis; grade 5 = four-leg paralysis; and grade 6 = death due to EAE [3, 22–24]. Six days after MOG injection, F3 or F3.olig2 cells (1 × 10^6^ cells in 2 mL saline/mouse) were intravenously transplanted. All protocols of animal experiments were approved by the Institutional Animal Care and Use Committee of Laboratory Animal Research Center, Chungbuk National University, and the experiments were conducted in accordance with the standard operation procedures.

### 2.3. Evaluation of Spinal Cord Injuries

Demyelination and axonal damage of the spinal cords were evaluated 50 days after EAE induction. Thoracic spinal cords were fixed in 10% neutral formalin, and paraffin-embedded sections (4 *μ*m in thickness) were stained with LFB. Myelin destruction and loss were examined under a light microscope for the fragmentation or pale (decreased) LFB staining.

### 2.4. Evaluation of Inflammatory Responses

Infiltration of T cells and macrophages in the spinal cords was confirmed by immunohistochemistry. The cryosections (30 *μ*m in thickness) of the spinal cords were incubated with anti-mouse CD3 (1 : 1,000, rat polyclonal, Serotec, Bicester, UK) and rat anti-mouse Mac3 (1 : 100, rat polyclonal, Pharmingen, San Diego, CA, USA) antibodies for 1 hour at room temperature. The sections were incubated with biotinylated secondary antibody overnight at 4°C, followed by avidin-biotin complex kit (Vector Laboratory, Burlingame, CA, USA), and developed with diaminobenzidine (Sigma-Aldrich, St. Louis, MO, USA). Inflammatory cells were counted under the field of ×200 of a light microscope.

### 2.5. Bio-Plex Assays for Central and Peripheral Cytokines

The spleen, thymus, and lymph nodes were weighed to evaluate responses of the immune system. To analyze cytokines related to the central and peripheral inflammatory responses, Th1 and Th2 cytokines from spinal cords and lymph nodes were measured using Bio-Plex cytokine assay kits (Bio-Rad, Richmond, CA, USA), according to the manufacturer's instructions. Briefly, anticytokine-conjugated beads were placed in 96-well microtiter plates and then removed by vacuum filtration. After then, samples, detection antibody, and streptavidin-PE were sequentially added and incubated for 30 min under mixing at 300 rpm. Final samples were immediately analyzed by a Bio-Plex array system, and cytokine concentrations were automatically calculated by Bio-Plex software (Bio-Rad).

### 2.6. Distribution, Maturation, and Differentiation of Transplanted Cells

In order to identify and analyze the distribution of transplanted cells in the spinal cords, cryosections were double-immunostained with antibodies specific to human mitochondria (hMito; 1 : 100, mouse monoclonal, Millipore, Molsheim, France) and/or Olig2 (1 : 100, rabbit polyclonal, Millipore). The sections were incubated with primary antibodies overnight at 4°C followed by secondary antibodies conjugated with Alexa Fluor 488 or 594 (1 : 200, Invitrogen, Cergy-Pontoise, France) for 2 hours at room temperature.

For the evaluation of maturation and differentiation of transplanted cells, spinal cord sections were double-immunostained with anti-hMito and anti-MBP (1 : 200, rabbit polyclonal, Millipore) for maturation to oligodendrocytes, anti-hMito and anti-NF (1 : 200, rabbit polyclonal, Millipore) for differentiation into neurons, or anti-hMito and anti-GFAP (1 : 200, rabbit polyclonal, Millipore) for differentiation into astrocytes [25].

### 2.7. Expression of Neurotrophic Factors

Spinal cords were homogenized in 10 volumes of 0.5 M Tris-HCl buffer (pH 6.8) and centrifuged at 13,500 rpm for 6 min at 4°C to obtain the supernatant. The spinal cord homogenate was denatured by boiling for 5 min at 95°C in the Tris buffer containing 10% sodium dodecyl sulfate (SDS) and 10% ammonium persulfate, separated by electrophoresis on a 10% SDS-polyacrylamide gel, and transferred onto a polyvinylidene difluoride membrane in 25 mM Tris buffer containing 20% methanol, 1% SDS, and 192 mM glycine. After blocking for 2 hours with 5% skim milk in Tris-buffered saline-Tween 20 (TBS-T; 20 mM Tris, pH 7.6, 137 mM NaCl and 0.1% Tween 20), the membrane was incubated with BDNF, NGF, CNTF, and LIF antibodies (1 : 500 in 1% bovine serum albumin; Santa Cruz Biotechnology, Delaware, CA, USA) for 2 hours at room temperature. After washing with TBS-T, the membrane was incubated with a secondary antibody with horseradish peroxidase (1 : 200 in 2% skim milk; Santa Cruz Biotechnology) for 2 hours at room temperature. The membranes were developed using electrochemiluminescence solution (Amersham Biosciences, Piscataway, NJ, USA).

### 2.8. Statistical Analysis

Data are presented as mean ± SEM. Statistical analyses of clinical scores and organ weights were performed by one-way analysis of variance (ANOVA), followed by post hoc Tukey's multiple-comparison test. The Mann-Whitney* U* test was employed to analyze cytokine concentrations and infiltrated T cell and macrophage counts. Significant difference between group comparisons was determined at *P* < 0.05.

## 3. Results

### 3.1. Effects on the Clinical Signs of EAE Mice

**Figure 1:**
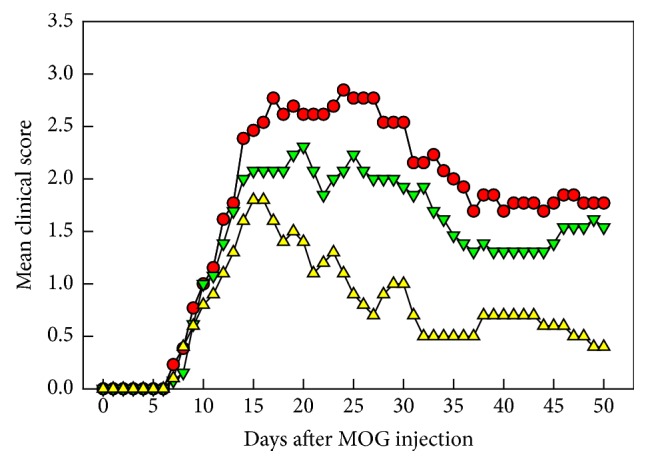
Effects of transplantation of F3 neural stem cells or F3.olig2 oligodendrocyte progenitor cells (1 × 10^6^ cells/mouse) on day 6 on the clinical abnormalities of experimental autoimmune encephalomyelitis (EAE) mice induced by myelin oligodendrocyte glycoprotein (MOG) on day 0. Red circle: MOG alone, green reverse triangle: MOG+F3 cells, and yellow triangle: MOG+F3.olig2 cells.

EAE mice revealed neurobehavioral deficits with mean onset time of 9.23 days after MOG challenge, reaching maximal scores 2.80 around 15–20 days after induction (Figure 1). Such symptoms were markedly attenuated by transplantation of F3 or F3.olig2 cells on day 6 to maximum scores 2.35 and 1.85, respectively, although the onset time was not affected. Notably, the clinical signs rapidly recovered in mice transplanted with F3.olig2 cells, leading to a significant decrease in the cumulative scores during 50-day observation period from 89.07 in vehicle-treated group to 37.80 in F3.olig2-injected mice that was superior to F3-treated animals (70.69).

### 3.2. Effects on the Spinal Cord Damage

**Figure 2:**
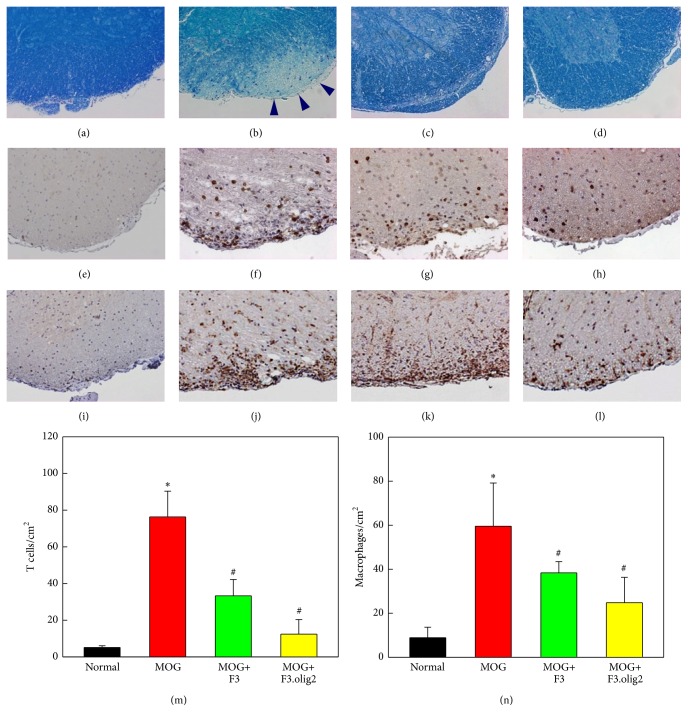
Representative findings of the spinal cords stained with Luxol fast blue ((a)–(d)) and immunostained with CD3 for T cells ((e)–(h)) or Mac3 for macrophages ((i)–(l)) and the numbers of T cells (m) and macrophages (n). (a), (e), and (i): normal, (b), (f), and (j): myelin oligodendrocyte glycoprotein (MOG) alone, (c), (g), and (k): MOG+F3 cells, and (d), (h), and (l): MOG+F3.olig2 cells; arrowheads: demyelinated area. ^*∗*^Significantly different from normal control (*P* < 0.05). ^#^Significantly different from MOG alone (*P* < 0.05).

MOG injection caused severe axonal shrinkage and demyelination of spinal cords, exhibiting markedly low intensity in Luxol fast blue (LFB) staining (Figure 2(b), arrowheads), compared with normal features in control animals (Figure 2(a)). Interestingly, however, transplantation of F3 cells remarkably attenuated the demyelination (Figure 2(c)). Better effects on the axonal and myelin alterations were achieved with F3.olig2 cells transplantation, exhibiting minimal axonal shrinkage and recovered intensity of LFB staining (Figure 2(d)), suggestive of the maintenance of axons and myelins.

### 3.3. Effects on the Inflammatory Response

The inflammatory progress, infiltration and activation, of peripheral T cells and macrophages into the spinal cords is related to negative effects of clinical signs as well as the severity of myelin and axonal pathology in EAE. In immunohistochemistry on CD3 for T cells and Mac3 for macrophages, the marked inflammatory cells infiltration was observed following immunization with MOG in the gray matter (Figures 2(f) and 2(j)), compared with the features of normal animals (Figures 2(e) and 2(i)). By comparison, the CD3 and Mac3 immunoreactivities were much less in the spinal cord gray matter of the animals treated with F3 or F3.olig2 cells, although F3.olig2 (Figures 2(h) and 2(l)) was superior to F3 transplantation (Figures 2(g) and 2(k)).

In a quantitative evaluation, the numbers of CD3-positive T cells and Mac3-positive macrophages increased in MOG-challenged EAE mice by 15-fold (76.0 cells/cm^2^) and 6-fold (60.0 cells/cm^2^) control levels (5.1 cells/cm^2^ for CD3 and 10.0 cells/cm^2^ for Mac3), respectively (Figures 2(m) and 2(n)). The MOG-induced inflammatory cells infiltration was significantly reduced by F3 or F3.olig2 cells, leading to a great anti-inflammatory effect of F3.olig2 cells.

### 3.4. Effects on the Inflammatory Cytokines

**Figure 3:**
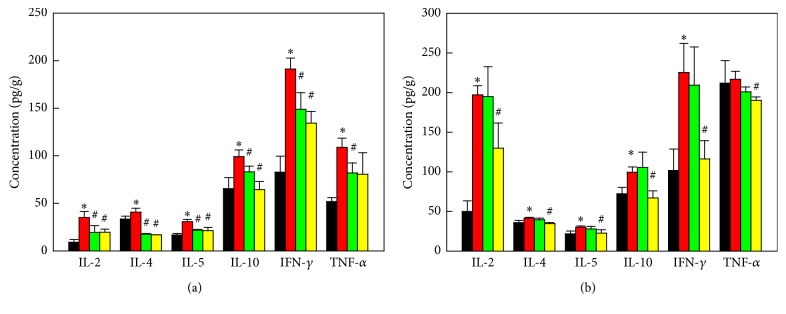
Effects of F3 or F3.olig2 cell transplantation on the myelin oligodendrocyte glycoprotein- (MOG-) induced increase in the cytokines in the spinal cords (a) and lymph nodes (b). Black: normal, red: MOG alone, green: MOG+F3 cells, yellow: MOG+F3.olig2 cells, IL: interleukin, IFN-*γ*: interferon-*γ*, and TNF-*α*: tumor necrosis factor-*α*. ^*∗*^Significantly different from normal control (*P* < 0.05). ^#^Significantly different from MOG alone (*P* < 0.05).

To elucidate immune-mediated inflammatory response as mechanisms, Th1/Th2 cell-mediated cytokines in spinal cords and peripheral lymph nodes were investigated by Bio-Plex assay. In both organs, production of various cytokines including interleukin-2 (IL-2), IL-4, IL-5, IL-10, and interferon-*γ* (IFN-*γ*), related to T cell activation, was markedly increased 50 days after challenge with MOG (Figure 3). Tumor necrosis factor-*α* (TNF-*α*) was also greatly increased in the spinal cord (Figure 3(a)), indicative of activation of inflammatory cells including macrophages. Both F3 and F3.olig2 cells downregulated the cytokines produced by T cells and macrophages in the spinal cord (Figure 3(a)), although F3.olig2 cells were more effective than F3 cells. Notably, F3.olig2 cells decreased the cytokines in the lymph nodes, while F3 cells were ineffective (Figure 3(b)). Particularly, F3.olig2 cells greatly downregulated Th1 cell-mediated cytokines such as IL-2 and IFN-*γ*, decreasing the ratio of Th1/Th2 (IFN-*γ*/IL-4), suggestive of a shift to Th2 response.

### 3.5. Distribution and Differentiation of Transplanted Cells

**Figure 4:**
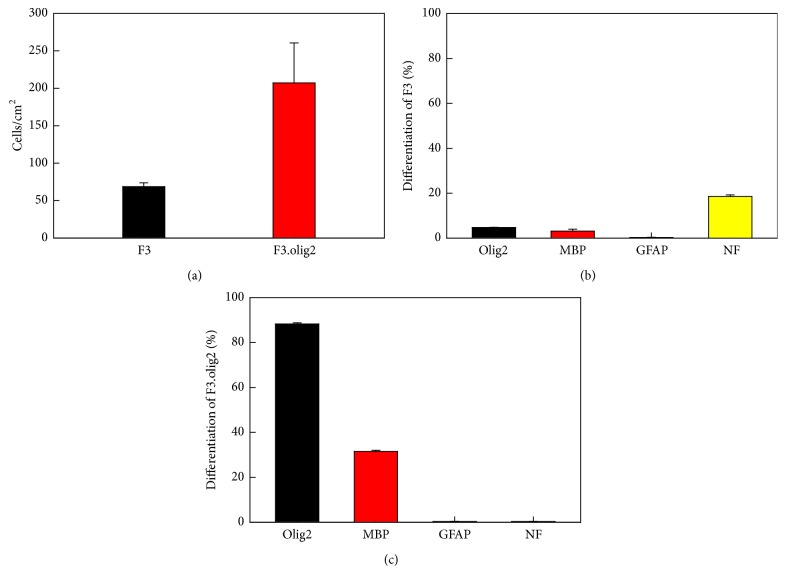
Numbers of transplanted human (hMito-positive) F3 and F3.olig2 cells in the thoracic spinal cords (a) and differentiation ratios of F3 (b) and F3.olig2 cells (c) in the myelin oligodendrocyte glycoprotein- (MOG-) challenged mice. Olig2: oligodendrocyte transcription factor, MBP: myelin basic proteins, GFAP: glial fibrillary acidic proteins, and NF: neurofilaments.

Forty-four days after intravenous transplantation of human F3 cells (1 × 10^6^ cells/mouse) to EAE mice, 69 cells/mm^2^ were observed in the thoracic spinal cord (Figure 4(a)). Notably, much more F3.olig2 cells (207 cells/mm^2^) were detected in the spinal cord, suggestive of a higher migratory activity of F3.olig2 cells than F3 cells. Notably, in the pattern of distribution, most of F3.olig2 cells were observed in the lesions, that is, in the gray matter of spinal cord and along the border of white and gray matters, while F3 cells were located around the lesion cavities or inside of white matter.

**Figure 5:**
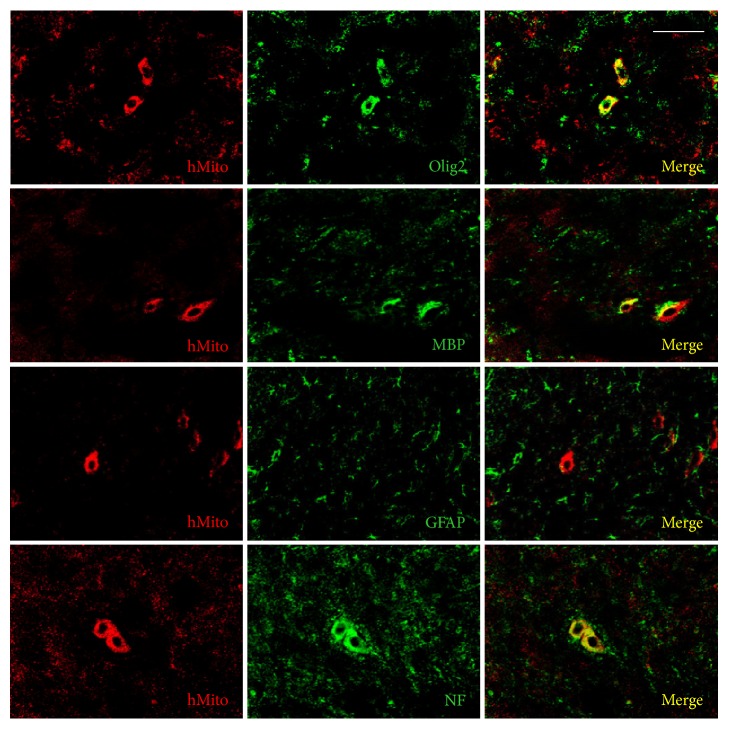
Differentiation of transplanted human (hMito-positive) F3 cells into oligodendrocytes (Olig2-positive), astrocytes (GFAP-positive), or neurons (NF-positive) and maturation to MBP-positive oligodendrocytes. Olig2: oligodendrocyte transcription factor, MBP: myelin basic proteins, GFAP: glial fibrillary acidic proteins, and NF: neurofilaments. Bar = 25 *μ*m.

**Figure 6:**
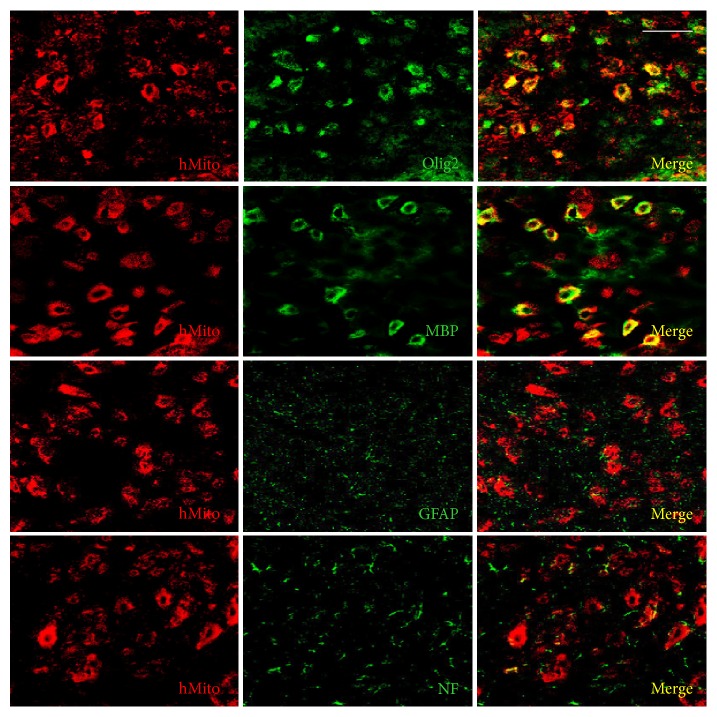
Differentiation of transplanted human (hMito-positive) F3.olig2 cells into oligodendrocytes (Olig2-positive), astrocytes (GFAP-positive), or neurons (NF-positive) and maturation to MBP-positive oligodendrocytes. Olig2: oligodendrocyte transcription factor, MBP: myelin basic proteins, GFAP: glial fibrillary acidic proteins, and NF: neurofilaments. Bar = 25 *μ*m.

Following intravenous transplantation of F3 human NSCs into the EAE mice, a part of the cells differentiated into oligodendrocytes (Olig2-positive) and maturated producing myelin basic proteins (MBP-positive) (Figure 5). The cells also differentiated into neurons [neurofilament- (NF-) positive], but not into astrocytes [glial fibrillary acidic protein- (GFAP-) positive]. Transplanted F3.olig2 oligodendrocyte progenitor cells also maturated and expressed MBP (Figure 6). In comparison with F3 NSCs, F3.olig2 cells did not differentiate into neurons or astrocytes.

In the ratio of differentiation, 18.55% of the transplanted human [human mitochondria- (hMito-) positive] F3 NSCs differentiated into NF-positive neurons in the brain environments, and only 4.75% of the cells differentiated into oligodendrocyte lineage (Olig2-positive) and 3.15% maturated to oligodendrocytes expressing MBP (Figure 4(b)). F3 NSCs did not differentiate into GFAP-positive astrocytes. By comparison, 90.50% of the transplanted F3.olig2 oligodendrocyte progenitor cells expressed Olig2, and 32.12% of them maturated (MBP-positive) (Figure 4(c)). Notably, F3.olig2 oligodendrocyte progenitor cells did not undergo reverse-differentiation process into neurons or astrocytes.

### 3.6. Modulation of Growth and Neurotrophic Factors Expression

**Figure 7:**
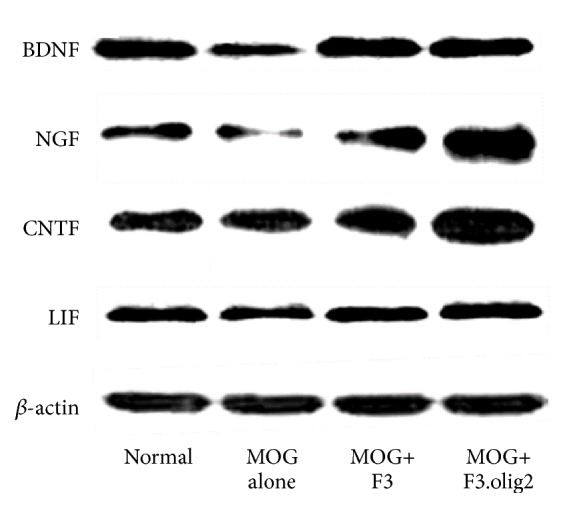
Effects of F3 or F3.olig2 cell transplantation on the myelin oligodendrocyte glycoprotein- (MOG-) induced change in the expression of growth and neurotrophic factors. BDNF: brain-derived neurotrophic factor, NGF: nerve growth factor, CNTF: ciliary neurotrophic factor, and LIF: leukemia inhibitory factor.

As additional mechanisms, growth and neurotrophic factors possessing immunomodulatory, neuroprotective, and neuroregenerative activities were measured. In the spinal cords of EAE mice, the expressions of brain-derived neurotrophic factor (BDNF), nerve growth factor (NGF), ciliary neurotrophic factor (CNTF), and leukemia inhibitory factor (LIF) decreased (Figure 7). However, the growth/neurotrophic factors were upregulated by transplantation of F3 and F3.olig2 cells, in which NGF and CNTF expressions were higher in F3.olig2-treated mice than in F3-transplanted animals.

## 4. Discussion

It is well known that stem cells and precursor cells improve MS symptoms via peripheral immunosuppression, leading to reduced central infiltration of inflammatory cells [3, 22, 26–28]. Thus, intravenous administration of neural precursor cells (NPCs) exerted a better effect on clinicopathology than intrathecal route [17]. However, stem cells are considered a promising therapy for the improvement of MS because of their neuroprotective and regenerative effects, in addition to the immunomodulatory activity. For example, neurospheres promoted multifocal remyelination [18], and adipose-derived mesenchymal stem cells (MSCs) increased the number of endogenous oligodendrocyte progenitors [22]. Bone marrow MSCs were also found to attenuate clinical signs of EAE not only by suppressing the peripheral B and T cells inducing Th1-polarized immune response, but also by promoting oligodendrogenesis [23, 29]. Such results indicate that precursor cells have advantages of lesion-tropic property after peripheral transplantation and myelin-generating potential following differentiation into oligodendrocytes. However, stem/precursor cells may have a limit to regenerate the damaged myelin sheath because only a part of the cells could differentiate into oligodendrocytes [14, 17, 22, 30, 31].

In the present study, NSCs (F3) and oligodendrocyte progenitor cells (F3.olig2: generated by transducing F3 cells with Olig2 transcription factor) were compared for their EAE-recovering activities. It was demonstrated that intravenous transplantation of both F3 and F3.olig2 cells attenuated the MOG-induced impairment of EAE animals. However, the therapeutic effect of F3.olig2 was much greater than that of F3 cells. The clinical scores, infiltration of peripheral inflammatory cells, and damage of spinal cord were markedly reduced in F3.olig2-transplanted animals.

Peripherally administered F3.olig2 cells may distribute and affect the lymphoid organs to suppress the activation of antigen-specific T cells and production of inflammatory cytokines like other NPCs [3]. Many studies showed that intravenously injected NPCs into the EAE mice were observed in the peripheral lymph nodes in a few days after injection, and the cells downregulated the proliferation and activation of T cells from the MOG-stimulated lymph nodes [3]. Notably, F3.olig2 cells significantly decreased the contents of lymph node cytokines which might be from activated T cells and macrophages. In contrast, F3 NSCs did not significantly suppress the peripheral cytokines, although it was also demonstrated that F3 suppressed T cell proliferation* in vitro* through the cell cycle arrest and induction of apoptosis [32]. Although underlying mechanisms remain to be clarified, the differences between neural stem cells and oligodendrocyte progenitors might be due to the altered signaling and interaction with inflammatory cells after differentiation and/or reciprocal stimulating or inhibiting effects on lymphocytes [19, 33]. Furthermore, it is of interest to note that adipose-derived MSCs differently affected the cytokines between early and late phases of EAE; that is, the stem cells downregulated both inflammatory and inhibitory cytokines probably by suppressing inflammatory cell activation and infiltration, but upregulated the inhibitory cytokines including IL-10 at late phase [22]. In the present study, it was also observed that the F3 NSCs and F3.olig2 cells administered at early phase downregulated all the cytokines analyzed in the spinal cord, which may be due to the inhibitory activity of the stem/progenitor cells on T cell and macrophage infiltration.

During MS development, peripherally autoaggressive T cells migrate into the CNS and finally induce the inflammation and demyelination [33]. However, resting T cells with the same antigen specificity cannot penetrate the blood-brain barrier (BBB) [34], indicating that peripheral activation of the inflammatory cells following priming by the antigen might be the prerequisite for the penetration across the BBB and reaction to the CNS antigens inducing their injury. Many stem cells including F3 NSCs have tumor- and lesion-tropic properties mediated by chemoattractants such as hepatocyte growth factor (HGF), stromal cell-derived factor-1 (SDF), vascular endothelial cell growth factor (VEGF), and stem cell factor (SCF) [25, 35]. Recent studies demonstrated that diverse growth/neurotrophic factors including BDNF and cytokines such as TNF-*α* and transforming growth factor-*β* (TGF-*β*) also trigger stem cell migration [36, 37]. Interestingly, more (4-fold) F3.olig2 cells than F3 cells, which were intravenously administered, migrated into the lesion area of the thoracic spinal cords, confirming a higher lesion-tropism of F3.olig2 progenitor cells than F3 NSCs. Notably, F3.olig2 cells suppressed the infiltration of T cells and macrophages as well as cytokine concentrations in the injured spinal cords, more effectively than F3 cells. Such effects might be due to their higher CNS-penetrating capacity and anti-inflammatory activity than NSCs, in addition the higher efficacy on the peripheral cytokines. Such high CNS-penetrating capacity of F3.olig2 cells was inferred from reports that intravenously injected NPCs exhibited an ability to pass through the BBB and moved into the demyelinated areas under lipopolysaccharide- (LPS-) and MOG-induced inflammatory conditions [18].

It is of interest to note that undifferentiated stem cells after locating into the demyelinated sites provide the proper environments for the stem cells to differentiate into the mature oligodendrocytes [38] and to contribute survival and self-renewal [24]. However, the previous and present studies showed that relatively low population of F3 NSCs migrated the damaged areas and differentiated into the myelin-producing oligodendrocytes* in vitro* and spinal cord injury rats [14]. That is, F3.olig2 progenitor cells maturated to MBP-expressing oligodendrocytes without reverse-differentiation into neurons and astrocytes, whereas F3 NSCs mainly differentiated into neurons, although only a small portion went to oligodendrocytes.

In spite of a very low differentiation of human NSCs into oligodendrocytes, EAE mobilizes neural progenitors from the subventricular zone to undergo oligodendrogenesis in adult mice [39]. Various studies reported that Olig2, as a key factor for the development of oligodendrocytes, provoked the activation of Nkx2.2 [14, 19, 20], and coactivation of Olig2 and Nkx2.2 induced oligodendrogenesis and remyelination in the spinal cords [40–43]. Furthermore, Olig2 gene in human NSCs promoted proliferation of the cells* in vitro* and* in vivo* [14]. These findings support that expressed Olig2 and activated Nkx2.2 in the F3.olig2 cells in the demyelinated sites might have stimulated the maturation of the cells into the oligodendrocytes, replacing the lesions and promoting myelin replacement.

It is well known that various growth and neurotrophic factors attenuate the EAE through the immunomodulatory, neuroprotective, and neuroregenerative effects [44, 45]. Furthermore, NSCs were found to increase directly and indirectly the neurotrophic factors under inflammatory conditions of CNS [6, 46, 47]. It was demonstrated that BDNF and NGF played key roles in neuronal and axonal survival and in enhancement of Th2 cell shift in EAE [44, 48, 49]. These immunomodulatory and neuroprotective activities of BDNF and NGF might have modulated the infiltration of T cells and macrophages and the production of inflammatory cytokines such as IFN-*γ* and IL-2 in the spinal cords, preventing the demyelination and axonal loss. CNTF and LIF have been shown to promote the survival of neurons and the differentiation of oligodendrocytes and to inhibit recruitment of T cells and macrophages into the CNS without affecting the peripheral immune system [45, 50, 51]. In addition, the growth/neurotrophic factors, especially BDNF, accelerate the proliferation and migration of grafted stem cells into the damaged areas [36]. F3 NSCs express high levels of growth and neurotrophic factors including BDNF, NGF, and CNTF [21], and transplantation of F3 and F3.olig2 cells fully recovered the decreased BDNF, NGF, CNTF, and LIF, suggesting that the increased growth/neurotrophic factors reduced the denuded axons in the spinal cords not only by alleviating the inflammatory demyelination and axonal degeneration, but also by stimulating regeneration of myelins.

In conclusion, our results revealed that F3.olig2 human oligodendrocyte progenitor cells exerted better beneficial effects in EAE mice than F3 NSCs through several mechanisms: suppression of the peripheral and central immunities as well as induction of neuroprotective growth and neurotrophic factors. Particularly the intravenously transplanted F3.olig2 cells migrated to the lesion area and maturated to myelinating oligodendrocytes, in comparison with a low differentiation of F3 cells into oligodendrocytes. The results indicate that F3.olig2 progenitor cells are superior to F3 NSCs in the improvement of EAE symptoms and that the F3.olig2 cells could be a good candidate for cell therapy of demyelinating diseases including MS.
